# Published trends and research hotspots of central venous catheter-associated thrombosis from 1973 to 2022: A scientometric analysis

**DOI:** 10.1097/MD.0000000000036111

**Published:** 2023-11-17

**Authors:** Zuoyan Liu, Xinxin Chen, Shiqi Tao, Jiuhong You, Hui Ma, Cheng Huang

**Affiliations:** a Department of Rehabilitation Medicine, West China Hospital, Sichuan University, Chengdu, Sichuan, China; b Key Laboratory of Rehabilitation Medicine in Sichuan Province, West China Hospital, Sichuan University, Chengdu, Sichuan, China; c West China School of Nursing, Sichuan University, Chengdu, Sichuan, China; d School of Rehabilitation Sciences, West China School of Medicine, Sichuan University, Chengdu, Sichuan, China; e Thoracic Oncology Ward, Cancer Center, West China Hospital, Sichuan University, Chengdu, Sichuan, China.

**Keywords:** central venous catheter, CiteSpace, intellectual mapping, scientometric analysis, thrombosis

## Abstract

This study aims to explore the intellectual landscape and research hotspots in the central venous catheter-related thrombosis (CVC-RT) research field. Studies discussing CVC-RT published from 1973 to 2022 in the Web of Science Core Collection database were retrieved on February 24th, 2022. Citespace was used to perform a scientometric analysis to identify the intellectual landscape and research hotspots in the research fields of CVC-RT. A total of 4358 studies were retrieved, with an ascending trend in publication numbers. The United States of America was the most influential country. The Journal of Vascular Access published the most studies, and McMaster University was the most prolific institution. The results showed that the focus population of CVC-RT research has changed from pediatric patients to cancer patients, the management of CVC-RT has become more formal and standardized, and the focused CVC type has shifted to port and peripherally inserted central catheters. In addition, seventeen active burst keywords were detected, such as patient safety, clinical practice guidelines, and postthrombotic syndrome. This study comprehensively reviewed publications related to CVC-RT. The research topics on patient safety, clinical practice guidelines, and postthrombotic syndrome related to CVC-RT may be future hotspots.

**Implications for clinical practice**

- The number of publications on central venous catheter-related thrombosis has increased in recent years, indicating that it is still a hot topic in clinical practice.
- The management of central venous catheter-related thrombosis has become more formal and standardized as many guidelines have been published. However, it is still necessary to update these guidelines and to facilitate their implementation in clinical practice.
- The topics of patient safety, peripherally inserted central catheters, home parenteral nutrition, and postthrombotic syndrome related to central venous catheter-related thrombosis require more medical staff attention in clinical practice.

## 1. Introduction

Central venous catheters (CVCs) are extensively used in clinical practice worldwide because of the advantages of providing long-term vascular access, administering irritant drugs and parental nutrition, and reducing puncture times. There are many types of CVCs whose tips target the junction of the superior vena cava and the right atrium, including CVCs inserted from the subclavian, jugular or femoral veins, peripherally inserted central catheters (PICCs), implantable venous access ports, and dialysis catheters.^[1,2]^ However, all types of CVCs can inevitably cause catheter-related complications, such as bloodstream infection (BSI), thrombosis, phlebitis, and infiltration,^[3–5]^ of which thrombosis is one of the most severe and common complications about which clinical staff are most concerned.^[5–7]^

CVCs can cause not only catheter-related thrombosis, which is generally defined as a mural thrombus extending from the catheter into the lumen of a vessel and resulting in partial or total catheter occlusion with or without symptoms^[8]^ but also deep vein thrombosis (DVT) and pulmonary embolism,^[9–13]^ indicating that CVC-related thrombosis (CVC-RT) is a potential life-threatening complication. The reported incidence of CVC-RT (including both symptomatic and asymptomatic CVC-RT) has varied significantly from 0.3% to 75% due to the differences in the definition of CVC-RT, study populations, CVC subtypes, study designs, precision of the diagnostic test used to detect CVC-RT, and other factors.^[8,9,13]^ It was reported that CVC-RT accounts for 10% of all DVTs in adults and 50% to 80% of all DVTs in children.^[14]^ CVC is the most common risk factor for venous thromboembolism (VTE) in children.^[15]^ Between 1994 and 2009, the reported incidence of hospitalized children increased significantly by 30% to 70%.^[16]^ CVC-RT was first reported in 1973, and a large number of studies on CVC-RT have been published at this point. Studies on CVC-RT have been carried out at all ages from infants to older adults^[4,7,17–19]^ and in different clinical settings, especially in oncology departments.^[8,20–23]^ Currently, insertion of CVCs has become a common practice in the oncology hematology department for systemic therapy.^[24]^ However, the occurrence of CVC complications has an important impact on the care-pathway. Studies have shown that the incidence of CVC-RT varies according to patient characteristics, catheter-related factors, and the steps involved in catheter insertion.^[12]^ Patients with cancer have a higher risk of thrombosis than the general population.^[24]^ Proper management of CVCs is a key element in the management of patients with cancer. With the widespread application of CVCs and the occurrence of thrombus complications, CVC-RT has attracted increasing attention, and the number of publications has increased significantly. The contents of CVC-RT studies include the interventions used to prevent and treat CVC-RT and the risk factors for CVC-RT.^[8,25–27]^ Therefore, it is urgent and necessary to systematically summarize the main findings and research trends of CVC-RT.

Bibliometric analysis is an analytical method involving mathematics and statistics that uses literature metrology characteristics to quantitively estimate the contribution of a specialized field.^[28]^ This method has been increasingly and widely used in many fields because it can help researchers detect research fronts and hot topics.^[29–32]^ In addition to describing and predicting future developments in a particular field of research, it can also compare the contributions of various authors, institutions, countries and journals. Although CVC-RT has received widespread attention for decades, few bibliometric studies related to the topic have been published. No bibliometric analysis has been performed on CVC-RT except for a metrological study on the 100 top-cited systematic reviews/meta-analyses in CVC, which showed that the second largest number of top 100-cited studies focused on CVC-RT following BSI.^[5]^ Bibliometric research rather than a traditional review is important in this high-profile topic because it can provide a visual summary of previous publications and predict potential frontiers. Well-organized bibliometric research can save time by selecting frontiers for researchers. Therefore, in this study, we aimed to perform bibliometric analysis of CVC-RT via CiteSpace to visually analyze the research trends and potential hotspots of CVC-RT and to provide references for future studies.

## 2. Methods

### 2.1. Data source and search strategy

We comprehensively searched the database of the Web of Science Core Collection to find studies related to CVC-RT from Jan 1, 1973, to Feb 24, 2022. The search strategy was as follows: TS = (central line OR central venous catheter OR central venous catheterization OR peripherally inserted central catheter OR femoral venous catheter OR venous port OR dialysis catheter) AND TS = (thrombus OR thrombosis OR thrombi OR thromboembolism). TS means a subject, which is contained by abstracts and keywords. We did not restrict the language, and we included only original articles and reviews.

### 2.2. Data collection

Two authors (Tao S.Q. and Liu Z.Y.) independently conducted the primary search in the database, and any discrepancy was resolved by discussion. To reduce the bias resulting from the frequent updates of the database of the Web of Science Core Collection, information on retrieved articles’ titles, abstracts, keywords, authors, counties, institutions, cited references, the number of publications, publication years, H-index of publications, annual citation, etc, were exported on February 24th, 2022.

### 2.3. Data analysis

The bibliometric analysis was carried out using CiteSpace 5.8. R3 software, as it is an optimal tool for collaborative network analysis connecting various publications.^[28,33,34]^ The following analyses were performed: analyses of collaborations among countries, institutions, and authors; keyword co-occurrence analyses; analyses of burst detection of keywords; and co-citation and cluster analyses of the included studies’ references and the timeline view of these clusters. The parameters of the CiteSpace software were set as follows: “year span” from 1973 to 2022 and “years per slice” of 5 years.

Nodes are set as a tree ring history, suggesting an article citation history or the publication volume of an object (country, journal, author, institution, keywords, etc). The sizes of the nodes or the nodes’ labels were positively correlated with the number of citations or the publication of objects. The links between nodes showed the presence of a co-occurrence or co-authorship relationship. The node colors indicate different years and gradually change from red to yellow. For example, red represents earlier years, and yellow represents recent years. A cluster is considered the embodiment of an underlying specialty. A cluster is considered to be reasonable when it shows an acceptable silhouette value that must be over 0.7. The silhouette value is an index used to evaluate the homogeneity of all members of a cluster.

### 2.4. Ethical considerations

This study was exempt from the Institutional Review Boards of West China Hospital Sichuan University because it was a bibliometric study not involving any human or animal experiments.

## 3. Results

### 3.1. Publication and citation trends in publications

**Figure 1.**
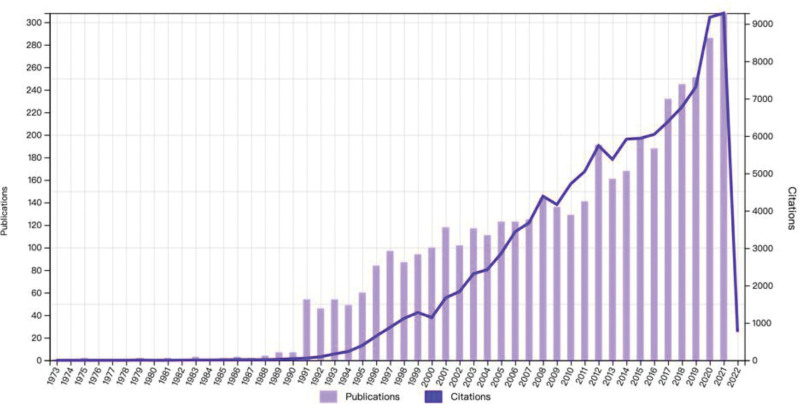
Trends in the publication and citation frequencies of the 4358 included articles from 1973 to 2022.

A total of 4358 articles were retrieved from the Web of Science Core Collection database. These articles cited a total of 53,907 references and were cited by 11,414 other articles. The first article on CVC-RT was published in 1973 by Fassoolt et al,^[35]^ titled “Prevention of concomitant thrombosis in central venous catheters by drugs,” and since then, the number of publications on CVC-RT has increased rapidly, especially after 1990. The number of citations also increased with time (Fig. 1).

### 3.2. Country, institution, and author co-occurrence analysis

All of the publications were from 87 countries, of which 87.81% were from the top 10 countries (Table 1). The United States of America (USA) published the most articles on CVC-RT (n = 1617), followed by Italy (n = 328), Canada (n = 327), and Germany (n = 326). The top 10 institutions that published the most articles were from either the USA or Canada. McMaster University from Canada ranked first. For authors, Chopra V was the most productive researcher, publishing 40 articles on CVC-RT.

**Table 1:** The top 10 countries, institutions, and authors publishing the most articles on CVC-RT.

Rank	Country	Count	Institution	Count	Author	Count
1	USA	1617	MCMASTER UNIV (Canada)	94	CHOPRA V	40
2	ITALY	328	UNIV TORONTO (Canada)	80	PITTIRUTI M	28
3	CANADA	327	UNIV MICHIGAN (USA)	65	FAUSTINO EVS	22
4	GERMANY	326	HOSP SICK CHILDREN (Canada)	61	BRANDAO LR	21
5	FRANCE	255	MAYO CLIN (USA)	51	CHAN AKC/MONAGLE P	20
6	ENGLAND	244	HARVARD UNIV (USA)	50	FLANDERS SA	19
7	PEOPLES R CHINA	231	JOHNS HOPKINS UNIV (USA)	39	TREROTOLA SO	18
8	THE NETHERLANDS	139	DUKE UNIV/UNIV TEXAS/UNIV WASHINGTON (USA)	36	ANDREW M/VAN OMMEN CH	16
9	AUSTRALIA	133	UNIV PENN(USA)	35	DEBOURDEAU P/GOLDENBERG NA	15
10	SPAIN	119	OHIO STATE UNIV (USA)	34	GOLDHABER SZ/GUNTHER RW/MASSICOTTE P/MOLINARI AC/RICKARD CM/STREIFF MB	14

### 3.3. Journal and co-cited journal analysis

As is shown in Table 2, the Journal of Vascular Access published the most articles on CVC-RT (n = 175), followed by the Journal of Vascular and Intervention Radiology (n = 109), Thrombosis Research (n = 81), and the Cochrane Database of Systematic Reviews (n = 62). In the top 10 journals, the Cochrane Database of Systematic Reviews has the highest H-index of 309. Journal co-citation analysis mainly focuses on analyzing the relevance and similarity between journals.^[36]^ The influence of a journal depends on its co-citation frequency, which reflects the influence of a journal in a specific research field. New England Journal of Medicine was the most co-cited journal, followed by Chest and Journal of Vascular and Interventional Radiology (Table 2). Among the top 10 journals, most were specialized journals that aimed to publish research on vascular access, interventional radiology, and vascular surgery, while half of the top 10 co-cited journals were comprehensive journals with high impact factors.

**Table 2:** The top 10 journals publishing the most articles on CVC-RT and the top 10 co-cited journals.

Rank	Journal	Count	IF^h^ 2022	CQ* 2022	H-index	Co-cited journal	IF^*^ 2022	CQ* 2022
1	J VASC ACCESS	175	2.326	Q3	42	NEW ENGL J MED	176.079	Q1
2	J VASC INTERV RADIOL	109	3.682	Q3	142	CHEST	10.262	Q1
3	THROMB RES	81	10.407	Q3	124	J VASC INTERV RADIOL	3.682	Q3
4	COCHRANE DB SYST REV	62	12.008	Q2	309	LANCET	202.731	Q1
5	J VASC SURG	60	4.190	Q2	210	THROMB HAEMOSTASIS	6.681	Q2
6	CARDIOVASC INTER RAD	55	8.271	Q1	92	RADIOLOGY	29.146	Q1
7	JPEN-PARENTER ENTER	55	3.896	Q3	110	J CLIN ONCOL	50.717	Q1
8	ANN VASC SURG	50	1.607	Q4	80	THROMB RES	10.407	Q3
9	J PEDIATR SURG	48	2.549	Q3	137	ANN INTERN MED	51.598	Q1
10	NEPHROL DIA TRANSPL	43	7.186	Q1	187	J VASC SURG	4.860	Q2

CQ = category quartile, IF = Journal Citation Reports Impact Factor.

### 3.4. Reference co-citation analysis

**Figure 2.**
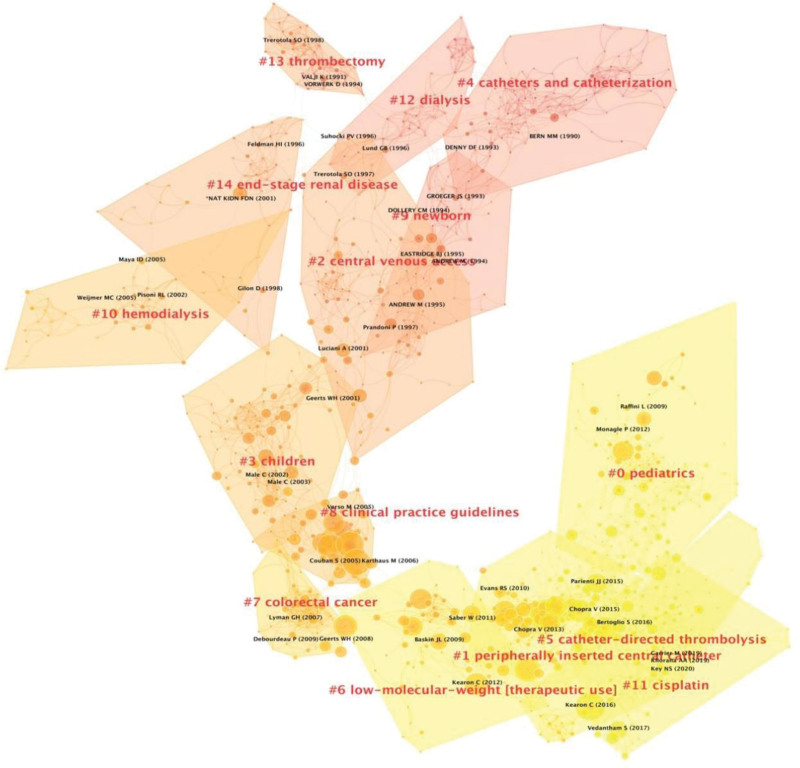
Reference co-citation network for the 4358 articles. (Link Retaining Factor = 3, Look Back Years = 5, and number of lines = 1.0). The cluster results were based on clustering the keywords. The colors of these clusters, moving from red to yellow, indicate citations from 1973 to 2022. The cluster blocks were distinguished by colors. Several circle nodes could be found in each cluster, and the sizes of the nodes indicated the times each reference had been cited. The lines among nodes represent the pathways of citation.

We conducted a cluster analysis of co-cited references of the 4358 included articles, and the landscape showed the top 15 cluster groups (Fig. 2) labeled by keywords. The 15 clusters reflecting the knowledge base and the developmen of the research in the CVC-RT field were #0 pediatrics, #1 peripherally inserted central catheter, #2 central venous access, #3 children, #4 catheters and catheterization; #5 catheter-directed thrombolysis, #6 low-molecular wight [therapeutic use], #7 colorectal cancer, #8 clinical practice guidelines, #9 newborn, #10 hemodialysis, #11 cisplatin, #12 dialysis, #13 thrombectomy, and #14 end-stage renal disease. The modularity Q value was 0.87, which can be considered very high, indicating that the specialties in the science mapping are clearly defined in terms of co-citation clusters. The silhouette values of these 15 clusters were all over 0.8, indicating that each cluster was highly homogeneous and reasonable.^[37]^ The different colors of the links and cluster areas represent the timeline transition; for example, cluster areas in red were generated earlier than areas in yellow.

**Figure 3.**
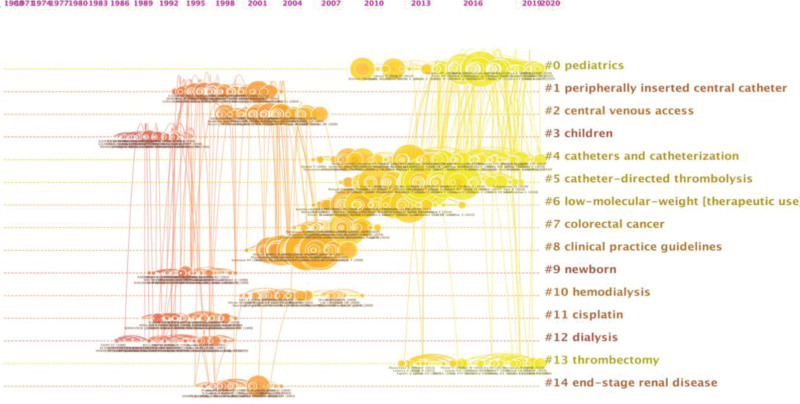
Timeline view of the citation trends identified in the 4358 included articles. (Link Retaining Factor = 3, Look Back Years = 5, and number of lines = 1.0). The timeline view map shows changes in reference co-citations over time. The circular areas represent citation tree rings, indicating the citation history of each article. Each node represents a cited reference, and the size of the node indicates the citation frequency. The color of the citation tree ring represents the year of publication. The cluster labeling on the right side indicates the research hotspot category related to each reference.

In addition, we adopted a timeline view of the cluster results to help us more clearly understand the evolution process of CVC-RT research over the years (Fig. 3). The earliest research direction lay in clusters #3, children, and #12, dialysis, with most publications dated approximately 1983. Clusters #0, pediatrics, #4, catheters and catheterization, #5, catheter-directed thrombolysis, and #13, thrombectomy, were the focus of the latest publications, dated approximately 2010.

### 3.5. Emerging trends and research frontiers of CVC-RT

#### 3.5.1. Keyword co-occurrence analysis.

**Figure 4.**
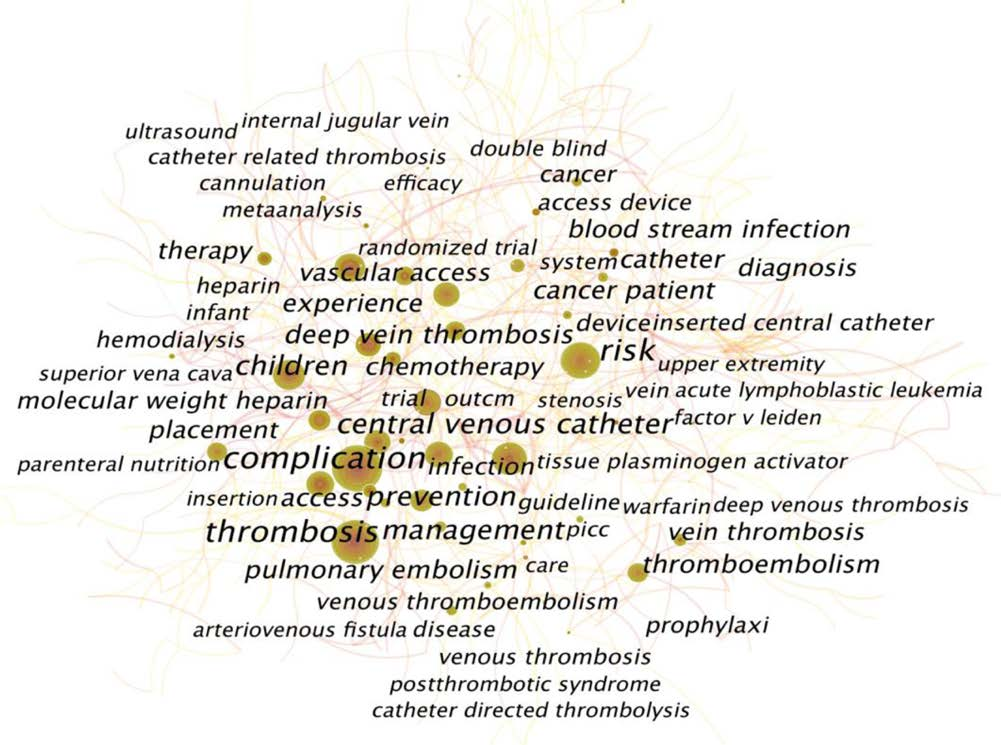
Keyword co-occurrence network map. This map only shows the keywords appearing in the 4358 included articles with high frequency. The higher the frequency is, the larger the sizes of the label and the node.

Keyword co-occurrence analysis is an excellent way to detect research trends and hotspots. As shown in Figure 4, the top 3 high-frequency co-occurrence keywords were “complication,” “risk,” and “thrombosis,” followed by “central venous catheter,” “prevention,” “management,” “children,” “deep vein thrombosis,” “thromboembolism,” “cancer patient,” “access,” “experience,” and “pulmonary embolism.” The thickness of the line between nodes indicates keyword collaboration. Most of the keywords were connected, for example, cancer and vascular access, children and central venous catheter, central venous catheter and guideline.

#### 3.5.2. Keyword burst detection analysis.

**Figure 5.**
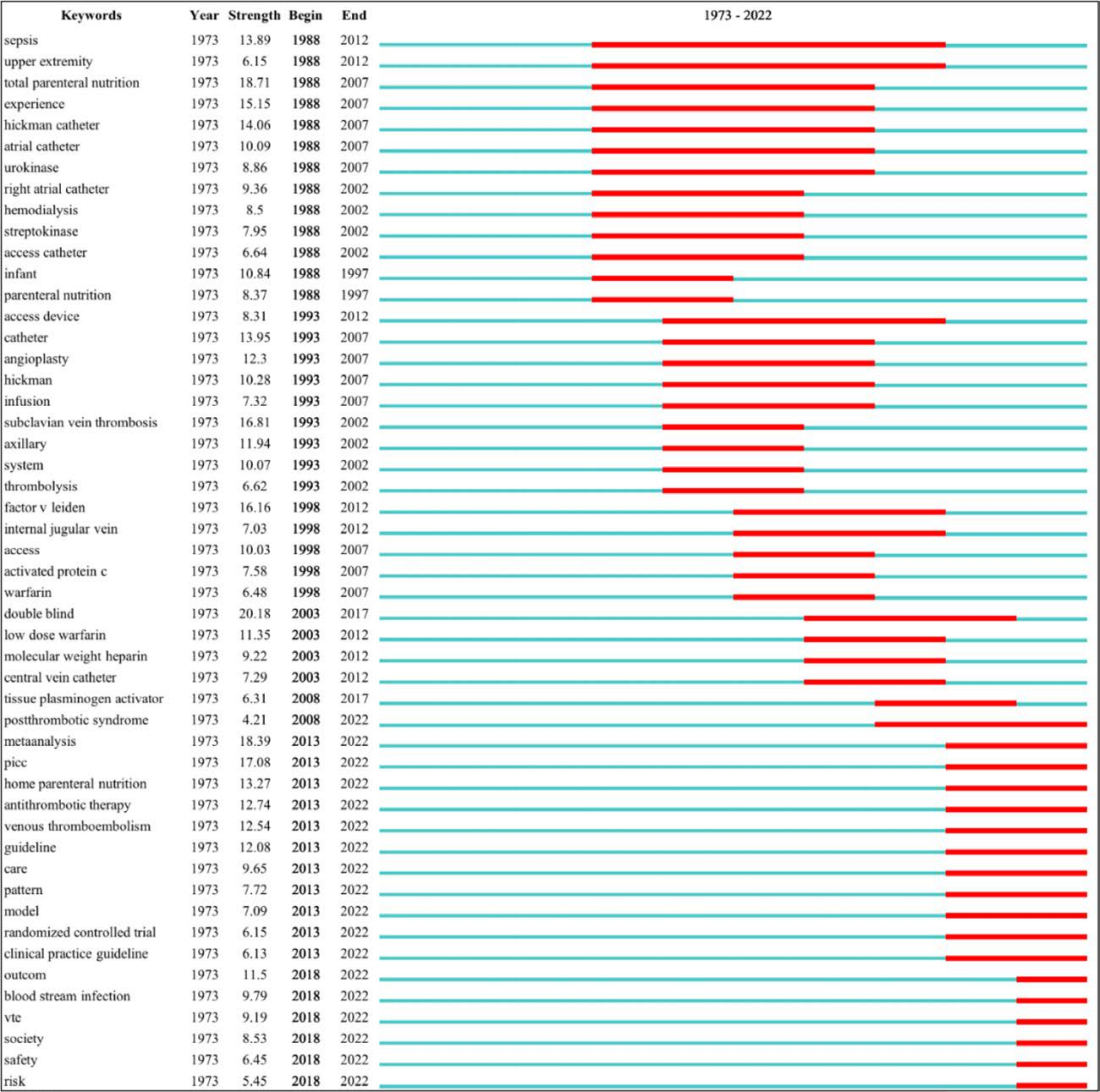
Time trends of burst keywords involved in the 4358 articles. In this figure, “Year” represents the time when the keywords first appeared; “Strength” represents the citation strength; and “Begin” and “End” represent the times when the keyword burst started and ended, respectively. The time span, from 1973 to 2022, is plotted on the blue line, while each period of keyword burst is highlighted in red.

Burst detection is an analysis method to detect whether there is any significant change, such as surges in publication and citations during a specific time span, and marks the corresponding time span in red. The burst strength indicates the growth rate of keywords being cited over time. The top 10 highest strength keywords were “double blind” (active from 2003 to 2017), “total parental nutrition” (active from 1988 to 2007), “metaanalysis” (active from 2013 to 2022), “picc” (active from 2013 to 2022), “subclavian vein thrombosis” (active from 1993 to 2002), “factor V leiden” (active from 1998 to 2012), “experience” (active from 1998 to 2007), “hickman catheter” (active from 1988 to 2007), “catheter” (active from 1993 to 2007), and “sepsis” (active from 1988 to 2012). The keywords that were still active included “metaanalysis,” “picc,” “inserted central catheter,” “antithrombotic therapy,” “venous thromboembolism,” “guideline,” “outcom,” “blood stream infection,” “care,” “thromboembolism,” “vte,” “society,” “pattern,” “model,” “safety,” “randomized controlled trial,” and “clinical practice guideline,” which may reflect the potential research frontier and hotspot. More details are shown in Figure 5.

## 4. Discussion

Thrombosis is one of the most severe and common complications of CVCs. In recent years, the studies on CVC-RT have been increasing, indicating that central venous catheter-related thrombosis is still a hot spot in clinical research. This research applied CiteSpace software to perform a bibliometric analysis of published articles relating to studies about CVC-RT. Firstly, annual publication number, journal, country, institution, author, and reference were quantitatively analyzed. We also summarized the research hotspots in the past years and speculated frontiers in the near future. The results showed that the research field of CVC-RT is quite active, as we can see even superficially from the number of published articles on CVC-RT, which indicated that there was an increasing trend in the number of publications in the past 2 decades. CVC-RT can affect the quality of life of patients and can even lead to death, but it is not well managed in clinical practice. Thus, it is still a challenging clinical problem that needs medical staff attention and further research.

**Figure 6.**
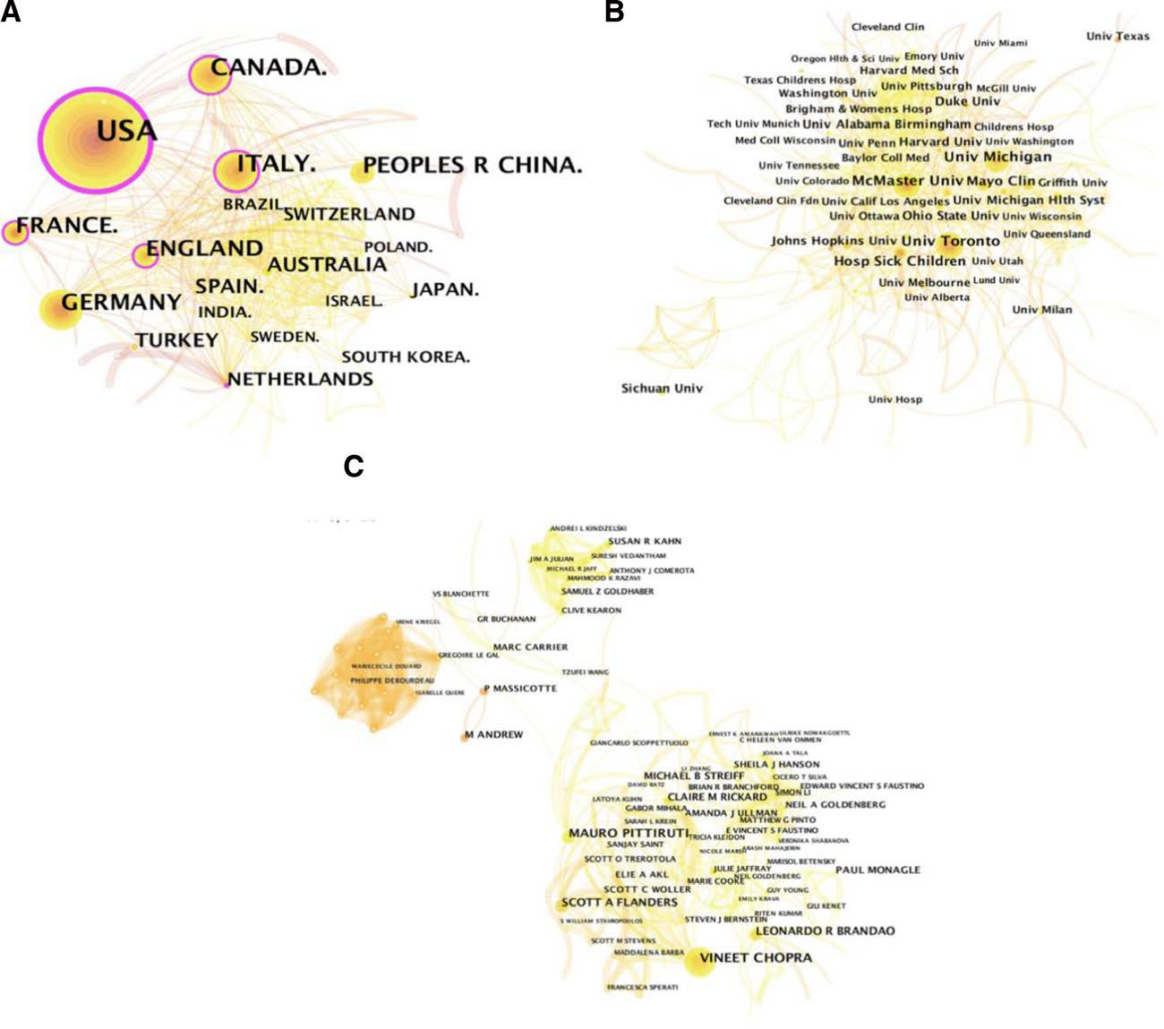
Cooperation networks among countries (A), institutions (B), and authors (C) on CVC-RT research. CVC-RT = central venous catheter-related thrombosis.

Collaboration mapping can be used to discover the social relationships among scholars, countries and institutions in a certain research field; it also provides a new perspective for evaluating the academic influence of scientific scholars, countries or institutions and helps us discover those researchers, countries and institutions worthy of attention.^[38]^ The USA plays a leading role in CVC-RT research, which is similar to our previous study in which the author from the USA published the most meta-analyses on CVC.^[5]^ In other medical research fields, such as caregivers,^[39]^ bariatric surgery,^[40]^ oncolytic viruses,^[41]^ and scoliosis,^[42]^ the USA is still the most prolific country. For the most prolific institutions, McMaster University and the University of Toronto from Canada ranked first and second, respectively; however, more than half of the top 10 institutions were from the USA, which also indicates the important contributions of the USA in CVC-RT research. Figure 6 shows that the top 10 countries, institutions and authors were prominent in the cooperation network and had extensive collaborations with other countries, institutions and authors. However, we could see only a few developing countries or institutions from developing countries in the cooperation network, indicating that there is a large gap between developing countries and developed countries in CVC-RT research.

Journal analysis of specific research fields could provide a reference for us to choose a proper journal before submission. In addition, to some extent, it can also reflect the disciplines involved in this research field. CVC is a technique providing vascular access that requires a multidisciplinary approach involving surgeons, interventional radiologists, anesthetists, and specially trained nurses.^[43]^ As shown in Table 2, we could easily find that among the top 10 journals publishing the most articles on CVC-RT and the top 10 co-cited journals, most journals were associated with surgery and radiology. In addition, 2 journals (*Thrombosis Research and Thrombosis Haemostasis*) targeted thrombosis. It is also noteworthy that the *Journal of Vascular Access,* which published the most articles on CVC-RT, is a journal established to publish articles on vascular access, especially on CVC, as it was estimated that approximately half of the articles published in this journal were related to CVC. In addition, many associations have been found to facilitate the development of CVC, such as the Association for Vascular Access, the Infusion Nursing Society, and the Australian Vascular Access Society. What is mentioned above indicates that the CVC research fields have gradually become mature and independent.

The reference co-citation clusters represent the intellectual base of specific research fields and show the evolution of the research field.^[28]^ Figures 2 and 3 show that the research focus has gradually changed in several aspects in the research field of CVC-RT. First, the focus population has changed from children and newborns to both pediatric patients and cancer patients. Previous studies reported that CVC is the most important cause of DVT in children and infants, and CVC-RT accounted for a large proportion of venous thromboembolism in children compared with adult patients.^[44–46]^ Therefore, many studies have focused on pediatric patients undergoing CVC-RT. Nonetheless, the incidence of venous thromboembolism in pediatric patients was notably lower than that in adult patients, especially for patients with cancer.^[12,47]^ In fact, there may be mechanisms (i.e., decreased capacity to generate thrombin and enhanced capacity to inhibit thrombin) in children to protect them from venous thrombosis.^[48–51]^ In the past few decades, many changes have been made in oncology, especially with respect to new chemotherapy schemes, that largely improve cancer patients’ survival rate. Patients with cancer frequently require repeated venipuncture for chemotherapy, therapy monitoring, parental nutrition, and other measures. With the advantages of reducing venipuncture frequencies, tolerating irritative and hyperosmolar infusions, and providing long-term vascular access, CVCs have been increasingly widely used in patients with cancer in recent years. According to data from the USA, at least 5 million CVCs are inserted annually.^[52]^ It is undeniable that CVCs play an important role in tumor treatment; however, they significantly increase the risk of DVT, which is considered an independent important prognostic factor for death and tumor progression.^[53,54]^ Moreover, cancer patients themselves are usually in a hypercoagulable state. A study reported that the synergistic effect of cancer with CVC could largely increase the risk of VTE by 43.6 times (adjusted OR, 95% CI: 25.5–74.6) after adjustments for sex and age.^[55]^ Therefore, it is urgent to carry out further research on CVC-RT in cancer patients, as there are still many controversies regarding the prophylaxis, treatment and management of CVC-RT in cancer patients.^[8]^ More high-quality studies on CVC-RT in pediatric patients are still needed since most of the evidence for children is drawn from low-quality studies or extrapolated from adult practice despite the huge differences between children and adults.^[17]^

Second, we found that the management of CVC-RT has become more formal and standardized, as clinical practice guidelines have emerged in recent years. The first practice guideline, especially for CVC-RT, was published in 2009,^[56]^ and the second guideline was published in 2013.^[8]^ Both guidelines are applicable to cancer patients, which also reflects the importance of solving the problem of CVC-RT in cancer patients. Two other guidelines targeting CVC include some content on CVC-RT.^[20,57]^ After reading these guidelines, we found that most evidence was drawn from low- to moderate-quality studies. Moreover, all of them were published nearly 5 years ago. However, in the last 5 years, many new studies on CVC-RT have been published.^[58–63]^ Therefore, it is necessary to update guidelines in a timely manner. In addition, several other guidelines have also included some recommendations for CVC-RT, such as the guidelines for the treatment and prophylaxis of venous thromboembolism in cancer patients,^[22,23]^ the guidelines for infusion therapy (2021),^[1]^ guidelines for the prevention of VTE in nonsurgical patients, guidelines on home parenteral nutrition (2020),^[64]^ and guidelines for the management of venous thromboembolism in pediatric patients (2018),^[17]^ but the recommendations on CVC-RT in these guidelines are not comprehensive, and much evidence is weak. Another concern is that there is always a delay in implementing these guidelines in clinical practice, probably owing to perceptions of clinical judgment as the primary element in clinical decision-making. Consequently, these facts could gradually weaken the credibility of guidelines and ultimately increase the difficulty of disseminating and implementing them. As shown in Figure 5, clinical practice guidelines are still a research hotspot. Therefore, the task may be not only to update guidelines on CVC-RT but also to disseminate and implement them at each national level.

Finally, the focus on CVC type has shifted to totally implantable venous access devices (ports) and PICCs. Compared with other types of CVCs, the ports have the advantages of a lower risk of catheter-related BSI and CVC-RT, providing very long-term vascular access (>3 months) and allowing patients to move freely, which greatly improves their quality of life. Even though the ports are quite expensive (at least 1000 dollars) in terms of insertion, maintenance, removal and complications, they are still extensively used, particularly in developed countries. It is estimated that approximately one million ports are implanted each year worldwide.^[52,65]^ Although PICCs have a higher risk of CVC-RT in critically ill or cancer patients than other types of CVCs,^[7]^ they can be inserted by specially trained nurses at the patient bedside, which makes their use more convenient and accessible in settings that may significantly facilitate their utilization in clinical practice.^[65–67]^ This could also explain why PICCs emerged as a research hotspot.

The keyword co-occurrence map could help us analyze the evolution of research hotspots, especially with keyword burst detection analysis. Figure 5 shows that new research hotspots have emerged, such as patient safety, clinical practice guidelines, postthrombotic syndrome (PTS), PICC, randomized controlled trial, home parenteral nutrition and bloodstream infection. Patient safety is prone to balancing the benefits of anticoagulation with the risk of major bleeding in clinical practice. The clinical practice guidelines, as we mentioned before, still need to be updated, disseminated and implemented in clinical practice. PTS is a chronic and potentially disabling complication caused by DVT, characterized by symptoms varying from mild edema to chronic pain and ulcers in the affected limbs.^[68]^ In children, the reported total incidence of PTS can be as high as 65%.^[12]^ However, CVC-related DVT was estimated to account for more than 50% of VTE cases in children.^[44,45]^ Even when the thrombus is initially asymptomatic, 8% to 18% of critically ill children could develop catheter-related PTS,^[69]^ which highlights the necessity to carry out further studies on PTS caused by CVC-RT in children. In adults, the reported incidence of PTS after upper-extremity DVT ranges from 7% to 46% due to the difference in the definition of upper-extremity PTS among these studies.^[70,71]^ However, a systematic review published in 2006 and another recently published original study suggested that upper-extremity CVC-RT was associated with a decreased risk of PTS. Since there is currently no gold standard to define and diagnose PTS, it is necessary to unify the definition and diagnostic criteria of upper-extremity PTS and to further validate whether CVC-RT is associated with a lower risk of PTS.

Home parental nutrition (HPN) is a basic life-saving treatment that is frequently used in patients with chronic intestinal failure (resulting from benign or malignant diseases) or patients in the late phase of end-stage disease, indicating that it is widely used[14]. Many clinical practice guidelines on patients with HPN have also been published,^[72–75]^ of which one guideline has been updated twice (2016 and 2020) since it was first published in 2009 (64, 73, 76). CVC, which is the primary way to deliver HPNs,^[64]^ requires medical staff attention considering the wide use of HPNs. CVC-RT in patients with HPN is a severe complication that can lead to the interruption of nutrition support and may be an indication for intestinal transplantation once 2 or more central venous vessels are affected by CVC-RT.^[76]^ The incidence of CVC-RT in patients with HPV was low, approximately 0.02 to 0.09 cases/catheter/year, as reported by several retrospective studies that focused only on symptomatic CVC-RT.^[77–82]^ One prospective observational study with a small sample size (n = 62) of participants with benign diseases reported that the incidence of CVC-RT was 0.045/catheter/year. It is worth noting that 41/62 patients received anticoagulant treatment during the observation period. Thus, we believe that the incidence of CVC-RT in patients with HPN is underestimated, especially for patients with cancer who need long-term CVCs (>1 year). In addition to the incidence of CVC-RT in patients with HPN, the prevention and treatment of CVC-RT in these patients are still controversial, even in the latest guidelines.^[64,75]^ The current evidence is weak because of the lack of prospective and large-sample studies on CVC-RT in patients with HPN. With the advancement of treatment of various diseases in recent years, the survival time of patients has been largely extended, especially for patients with cancer, which means that there may be huge underlying patient populations needing HPN both at present and in the future. Therefore, it is essential and necessary to carry out more prospective, large-sample, multicenter studies in the future to explore the incidence and patterns of CVC-RT, the long-term effects of CVC-RT, and the safety and efficacy of thromboprophylaxis and treatment in patients with HPN.

## 5. Limitations

This study also has some limitations. First, we included only data from the Web of Science Core Collection Database because the software CiteSpace supports the analysis of only the data from this English database. Further studies can be extended to analyze the data from other databases. Second, we selected only the literature published before February 24th, 2022. However, the database is updated continuously; thus, this study missed the studies published after the day we downloaded the data.

## 6. Conclusion

In this study, we first analyzed the publication information of CVC-RT-related studies from 1973 to 2022. The results showed that there was an increasing trend over the past years; the USA was the most influential country, the *Journal of Vascular Access* published the most studies, and McMaster University was the most prolific institution. Second, we analyzed the intellectual base and the evolution of the CVC-RT research field. The reference co-citation analysis resulted in fifteen clusters, and we found that the management of CVC-RT became more formal and standardized, and the focused CVC type shifted PICCs. Finally, we analyzed the research hotspots of CVC-RT. The results showed that several potential research hotspots emerged, such as the validation of the relationship between PTS and CVC-RT, the update and implementation of clinical practice guidelines on CVC-RT, and how to better manage PICC-related thrombosis. Further high-quality studies are needed to better prevent and treat CVC-RT.

## Author contributions

**Conceptualization:** Zuoyan Liu, Xinxin Chen, Cheng Huang.

**Formal analysis:** Zuoyan Liu.

**Funding acquisition:** Cheng Huang.

**Investigation:** Cheng Huang.

**Writing – original draft:** Zuoyan Liu, Xinxin Chen, Shiqi Tao, Jiuhong You, Hui Ma.

**Writing – review & editing:** Zuoyan Liu, Xinxin Chen, Shiqi Tao, Jiuhong You, Hui Ma, Cheng Huang.
